# EMOTION REGULATION IN PEOPLE LIVING WITH DEMENTIA AND THEIR SPOUSES: THE ROLE OF NEUROPSYCHIATRIC SYMPTOMS

**DOI:** 10.1093/geroni/igae098.2690

**Published:** 2024-12-31

**Authors:** Yifan Lou, Joan Monin, Thi Hoang Vu, Amanda Piechota

**Affiliations:** Virginia Commonwealth University, New Haven, Virginia, United States; Yale School of Public Health, New Haven, Connecticut, United States; Yale University School of Public Health, New Haven, Connecticut, United States; University of Alabama, Tuscaloosa, Alabama, United States

## Abstract

People with dementia (PwD) and their care partners (CP) may have difficulties in emotion regulation and individual differences in emotion regulation may be related to PwD’s neuropsychiatric symptoms. This study explores whether there is any self-awareness of the PwD’s emotional behaviors and whether CP’s emotion regulation relates to the PwD’s neuropsychiatric symptoms, potentially revealing bias or interpersonal effects. We used data from the Wish Outcome Obstacle Plan Study with a sample of 45 PwD and their spousal CP (n = 90 individuals). Multivariate linear regression models were used to investigate the associations between the CP-reported neuropsychiatric symptoms in PwD and self-reports of emotion regulation in both dyad members, net of sociodemographic and health factors. Separate analyses were conducted for each neuropsychiatric subsyndrome and each domain of difficulties in emotion regulation. Increasing numbers of neuropsychiatric symptoms were associated with higher difficulties in emotion regulation in PwD (ß = 2.78, p < 0.01), but not with CP’s perceived difficulties in emotion regulation. PwD reported particular difficulties in accepting emotions, controlling impulses, and accessing emotion regulation strategies, but not in emotion awareness and clarification, when CP reported more neuropsychiatric symptoms. Proxy-report hyperactivity and psychosis subsyndromes are most significantly related to PwD’s perceived difficulties in emotion regulation. PwD may be aware of their difficulties in emotion regulation at the early stage of dementia. Proxy-report neuropsychiatric symptoms may capture PwD’s emotion regulation capability and not be biased by CP’s emotion regulation difficulties.

